# Seasonal variations in cellular and humoral immunity in male striped hamsters (*Cricetulus barabensis*)

**DOI:** 10.1242/bio.038489

**Published:** 2018-11-07

**Authors:** De-Li Xu, Xiao-Kai Hu, Yufen Tian

**Affiliations:** 1 College of Life Sciences, Qufu Normal University, Qufu 273165, Shandong Province, China; 2 Library, Qufu Normal University, Qufu 273165, Shandong Province, China

**Keywords:** Body fat, Corticosterone, Glucose, Humoral immunity, Leptin, Phytohaemagglutinin response, Striped hamsters (*Cricetulus barabensis*)

## Abstract

Animals in the non-tropical zone usually demonstrate seasonal variations in immune function, which is important for their survival. In the present study, seasonal changes in immunity in striped hamsters (*Cricetulus barabensis*) were investigated to test the winter immunoenhancement hypothesis. Male hamsters were captured from the wild in the fall and winter of 2014 and in the spring and summer of 2015. Body mass, body fat mass and blood glucose levels of the hamsters were all highest in the summer, whereas relative fatness and thymus mass had no seasonal changes. Spleen mass was highest in the fall and white blood cells and phytohaemagglutinin (PHA) response indicative of cellular immunity were lowest in the summer among the four seasons, which supports the winter immunoenhancement hypothesis. IgG and IgM titers were lowest in the fall, which was against this hypothesis. Body fat mass had no correlations with cellular and humoral immunity, suggesting it was not the reason for seasonal changes in cellular and humoral immunity in males. Leptin titers were higher in spring and summer than in fall and winter. No correlation between leptin and cellular and humoral immunity suggested that leptin did not mediate their seasonal changes. Similarly, corticosterone levels were also higher in spring and summer than in fall and winter, which correlated negatively with cellular immunity but positively with IgG levels. This result implied that corticosterone has a suppressive effect on cellular immunity and an enhancing effect on humoral immunity. In summary, distinct components of immune systems exhibited different seasonal patterns.

This article has an associated First Person interview with the first author of the paper.

## Abstract (precis)

**Summary:** Seasonal changes in immunity, body fat, blood glucose, leptin and corticosterone and their correlations in striped hamsters.

## INTRODUCTION

Animals in the non-tropical area face seasonal changes in environment, hence seasonal variations are often observed in their many physiological processes, including immune responses, which protect them from infection and the attack of pathogens (Sheldon and Verhulst, 1996; Owens and Wilson, 1999; Nelson et al., 2002). According to the winter immunoenhancement hypothesis, immune function tends to be enhanced actively to counteract the immunosuppressive effects of stressors that occur in winter such as low ambient temperatures and reduced food availability (Nelson and Demas, 1996; Sinclair and Lochmiller, 2000; Nelson, 2004; Martin et al., 2007). This hypothesis is supported by some field and laboratory research. For instance, immune responses are higher in fall and winter than in spring and summer in common voles (*Microtus arvalis*) (Dobrowolska et al., 1974; Dobrowolska and Adamczewska-Andrezjewska, 1991), cotton rats (*Sigmodon hispidus*) (Lochmiller et al., 1994), red (*Clethrionomys rutilus*) and bank (*Clethrionom glareolus*) voles (Moshkin et al., 1998), prairie voles (*Microtus ochrogaster*) (Sinclair and Lochmiller, 2000) and Mongolian gerbils (*Meriones unguiculatus*) (Zhang and Wang, 2006). Similarly, several laboratory studies have indicated that immune enhancement can be induced by short days (Nelson and Demas, 1996; Brainard et al., 1987; Drazen et al., 2002; Bilbo et al., 2002). However, this hypothesis is not supported by other field research including in field voles (*Microtus agrestis*) (Newson, 1962), whose spleen mass was heavier in the summer than in the winter, and rhesus monkeys (*Macaca mulatta*) whose lymphoid cells had a greater potential to synthesize the proinflammatory cytokines during the summer than during the winter (Mann et al., 2000). It is also not supported by some laboratory research, in which short day-length reduced T cell-dependent antibody titers in Siberian hamsters (*Phodopus sungorus*) compared with long day-length (Yellon et al., 1999; Drazen et al., 2000). Therefore, further research is required to test this hypothesis in more species.

The adaptive immune system in vertebrates includes cellular and humoral immunity. The former is usually assessed by phytohaemagglutinin (PHA) response, which generally controls intracellular pathogens (Smits et al., 1999; Goüy de Bellocq et al., 2006; Xu et al., 2017). The latter, which is mainly responsible for extracellular pathogens, is often evaluated by measuring antibody production in response to a particular antigen (i.e. keyhole limpet haemocyanin, KLH) (Demas et al., 2003; Zysling and Demas, 2007; Zysling et al., 2009). The thymus is the main site of primary T cell development, and the spleen also plays an important role in immunity (Savino and Dardenne, 2000; Calder and Kew, 2002; Smith and Hunt, 2004). Moreover, white blood cells are involved in fighting against pathogens and hence are usually used to reflect the health status (Calder and Kew, 2002).

Leptin is a cytokine-like hormone secreted mainly by white adipose tissues, which serve as energy reserves, endocrine and immune organs (Zhang et al., 1994; Pond, 1996; Ahima and Flier, 2000; Trayhurn, 2005; Fantuzzi, 2005; Schäffler et al., 2007). Besides its regulatory role in energy balance, leptin is also important for regulating immune responses (Fantuzzi and Faggioni, 2000; Matarase et al., 2005; Lam and Lu, 2007; Lago et al., 2007). The hypothalamic-pituitary-adrenal axis is often activated by stressors and hence leads to the increase of stress hormones such as corticosterone, which usually has a suppressive influence on immune function (Sapolsky et al., 2000; Webster Marketon and Glaser, 2008).

Striped hamsters (*Cricetulus barabensis*) live mainly in northern China and they are also distributed in Russia, Mongolia and Korea (Zhang and Wang, 1998). The behavior of this species is granivorous, nocturnal and solitary. They eat stems and leaves of plants during summer and crop seeds in winter (Lu et al., 1987; Zhang and Wang, 1998; Song and Wang, 2003). Sandy areas, farmlands and grasslands are their favorite habitats. Hamsters often dig burrows in high dry areas to avoid the rain (Zhang and Wang, 1998; Song and Wang, 2003). The climate is arid, which is warm and dry in summer (the highest temperature is 42.6°C) and cold in winter (the lowest temperature is below −20°C). Therefore, striped hamsters are confronted with large seasonal changes in temperature, day length and food resources (Zhang and Wang, 1998; Zhao et al., 2010). Investigating seasonal variation in immunity in this species can help us to understand their immune adaptive strategies to seasons. In the present study, we tested the winter immunoenhancement hypothesis and expected that cellular and humoral immunity would be higher in fall and winter than in other seasons in male striped hamsters.

## RESULTS

### Body condition

**Fig. 1.**
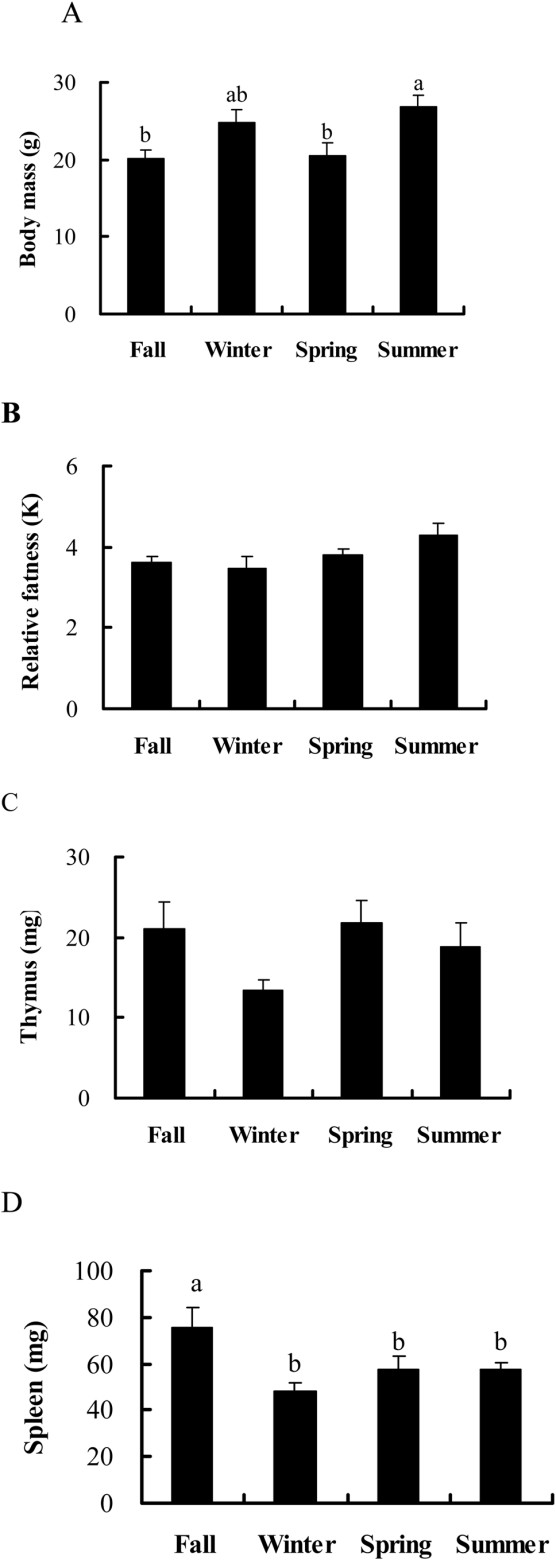
Seasonal changes of (A) body mass, (B) relative fatness (K), (C) thymus and (D) spleen mass in male hamsters.

Body mass was highest in the summer and lowest in fall among the four seasons in male striped hamsters (*F*_3,44_=4.332, *P*=0.009) (Fig. 1A). However, no seasonal change was observed in the relative fatness in male hamsters (*F*_3,44_=2.246, *P*=0.096) (Fig. 1B). Wet thymus mass (*F*_3,43_=1.877, *P*=0.148) did not show seasonal variation, while wet spleen mass was highest in the fall (*F*_3,43_=6.151, *P*=0.001) (Fig. 1C,D). From fall in 2014 to summer in 2015, wet carcass mass, subcutaneous fat, retroperitoneal fat, perigonadal fat, total body fat mass and their corresponding fat contents increased significantly, while there were no seasonal differences in mesenteric fat mass and its fat content in male hamsters (Table 1).

**Table 1.**
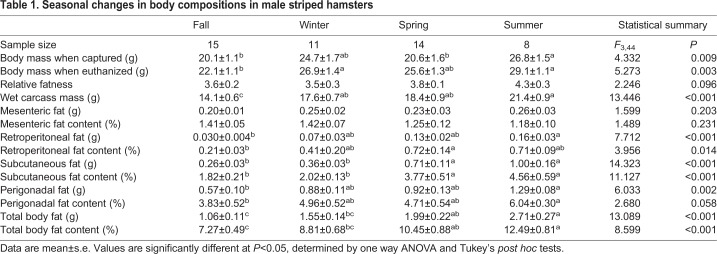
**Seasonal changes in body compositions in male striped hamsters**

### Immune responses

**Fig. 2.**
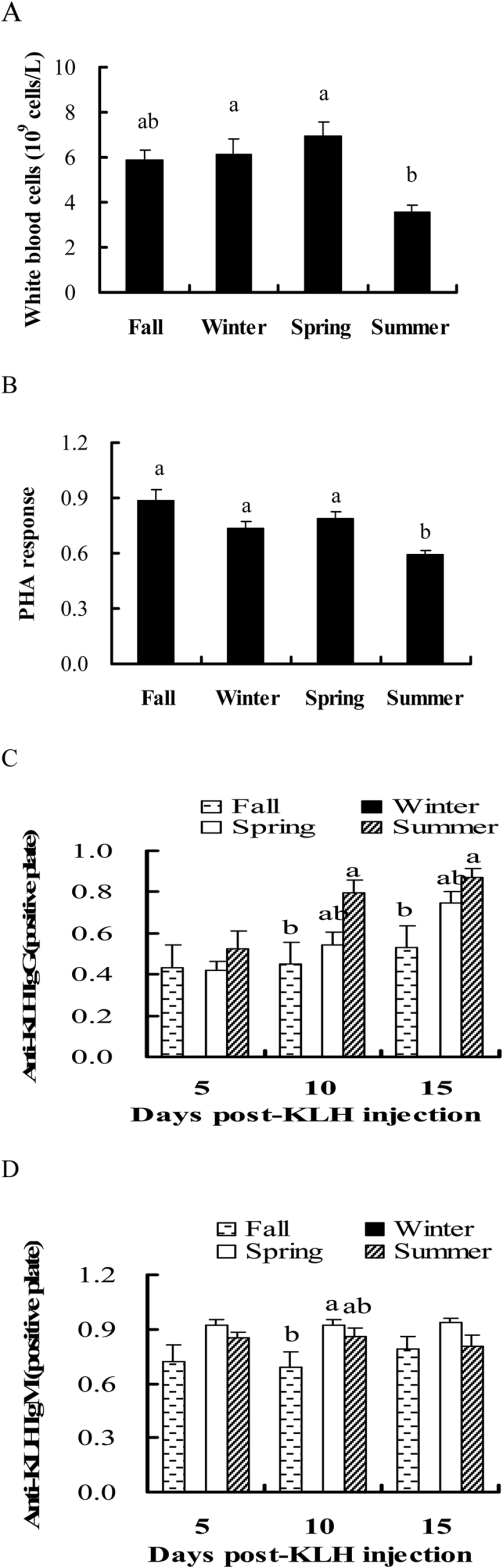
Seasonal changes of (A) white blood cells, (B) PHA response, (C) IgG levels and (D) IgM levels in male hamsters.

The number of white blood cells was lowest in the summer in male hamsters (*F*_3,44_=4.649, *P=*0.007) (Fig. 2A). Minimum PHA response occurred in the summer in male hamsters (*F*_3,44_=10.732, *P*<0.001) (Fig. 2B). It was negatively correlated with total body fat mass (r=−0.342, *P*=0.017) and relative fatness (r=–0.284, *P*=0.050) (Fig. S2A).

IgG titers after 10 (*F*_3,48_=3.397, *P=*0.045) and 15 (*F*_3,48_=4.062, *P*=0.026) days of KLH injection were the highest in summer of fall, spring and summer, whereas IgG titers post-5 day KLH injection had no seasonal changes (*F*_3,48_=0.301, *P=*0.742) (Fig. 2C). No correlation was observed between total body fat mass and IgG levels after 5 (r=−0.055, *P*=0.745), 10 (r=0.109, *P*=0.520) and 15 (r=0.114, *P*=0.503) days of KLH injection (Table S1).

IgM titers after 10 (*F*_3,44_=4.105, *P=*0.025) days of KLH injection were the lowest in the fall among the three seasons, while IgM concentrations after 5 (*F*_3,44_=2.628, *P=*0.087) and 15 (*F*_3,44_=2.114, *P=*0.136) days of KLH injection had no seasonal variation (Fig. 2D). Total body fat mass was not correlated with IgM titers after 5 (r=0.192, *P*=0.254), 10 (r=0.230, *P*=0.171) and 15 (r=0.110, *P*=0.515) days of KLH injection (Table S1).

### Blood glucose

**Fig. 3.**
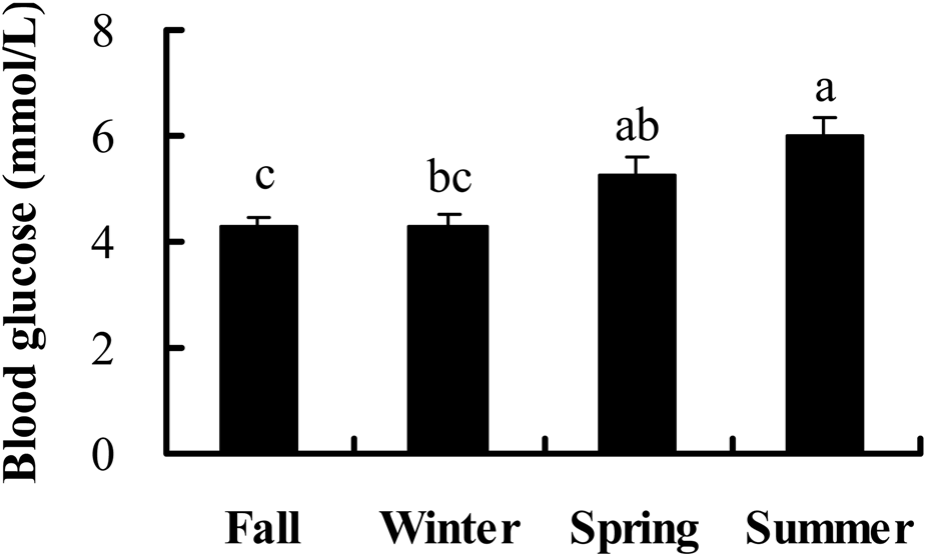
**Seasonal changes of blood glucose levels in male striped hamsters.** Different letters above the column indicate significant differences at *P*<0.05.

Maximum blood glucose levels were observed in the summer (*F*_3,44_=8.248, *P*<0.001) (Fig. 3). Blood glucose levels were positively correlated with total fat mass (r=0.392, *P*=0.006), but negatively with PHA response (r=−0.345, *P*=0.016) and not correlated with IgG levels after 5 (r=0.070, *P*=0.681), 10 (r=0.240, *P*=0.152) and 15 (r=0.268, *P*=0.109) days of KLH injection or IgM levels after 5 (r=0.011, *P*=0.950), 10 (r=0.046, *P*=0.786) and 15 (r=-0.109, *P*=0.522) days of KLH injection (Fig. S2, Table S1).

### Serum leptin concentration

**Fig. 4.**
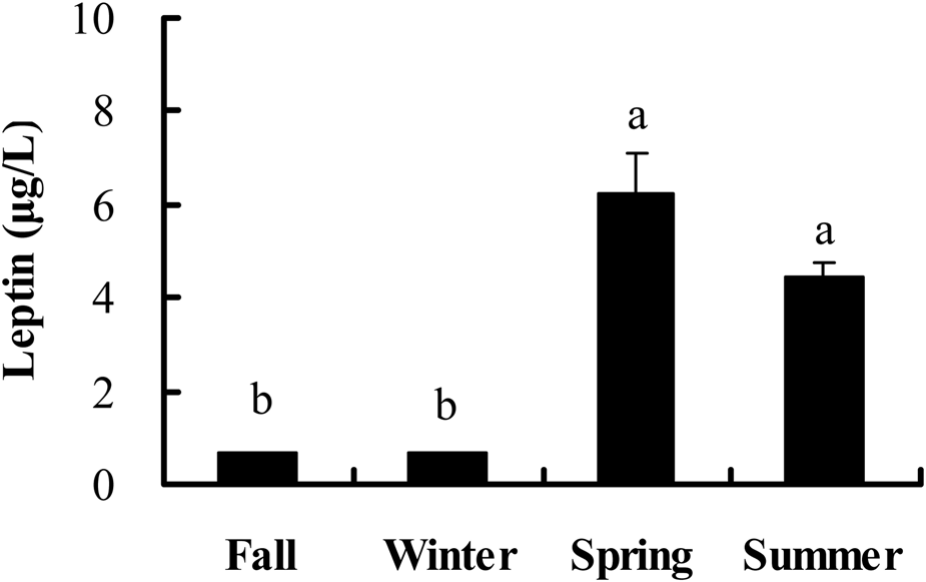
**Seasonal changes of leptin levels in male striped hamsters.** Different letters above the column indicate significant differences at *P*<0.05.

Leptin titers were higher in spring and summer than in fall and winter (*F*_3,44_=32.380, *P*<0.001) (Fig. 4). Leptin levels were positively correlated with body fat mass (r=0.548, *P*<0.001), glucose levels (r=0.575, *P*<0.001) and IgM levels after 10 days (r=0.339, *P*=0.043) of KLH injection. No significant correlation was observed between leptin levels and body mass (r=0.200, *P*=0.179), PHA response (r=−0.256, *P*=0.083) and IgG levels after 5 (r=0.100, *P*=0.564), 10 (r=0.128, *P*=0.458) and 15 (r=0.309, *P*=0.067) days of KLH injection, or IgM titers after 5 (r=0.315, *P*=0.061) and 15 (r=0.275, *P*=0.104) days of KLH injection (Table S1).

### Serum corticosterone titers

**Fig. 5.**
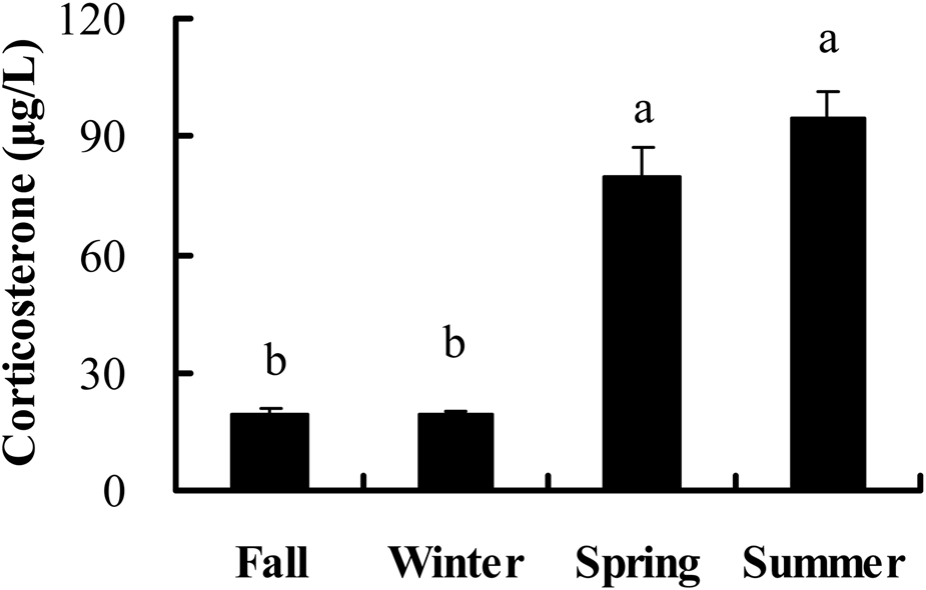
**Seasonal changes of corticosterone levels in male striped hamsters.** Different letters above the column indicate significant differences at *P*<0.05.

Corticosterone titers were higher in spring and summer than in fall and winter (*F*_3,44_=49.972, *P*<0.001) (Fig. 5). In the fall, corticosterone levels were positively correlated with IgG levels after 10 (r=0.551, *P*=0.033) and 15 days (r=0.554, *P*=0.032) of KLH injection, but not correlated with PHA response (r=0.047, *P*=0.867), IgG levels after 5 days (r=0.459, *P*=0.085), IgM levels after 5 (r=−0.483, *P*=0.068), 10 (r=−0.490, *P*=0.063) and 15 (r=−0.363, *P*=0.184) days of KLH injection. In the winter, there was no significant correlation between corticosterone levels and PHA response (r=0.024, *P*=0.945). In the spring, no significant correlations were observed between corticosterone levels and PHA response (r=−0.024, *P*=0.935), IgG levels after 5 (r=−0.010, *P*=0.974), 10 (r=0.066, *P*=0.822), 15 (r=−0.116, *P*=0.692) days, IgM levels after 5 (r=−0.180, *P*=0.539), 10 (r=−0.435, *P*=0.120) and 15 (r=0.102, *P*=0.728) days of KLH injection. In the summer, corticosterone levels had no correlation with PHA response (r=0.086, *P*=0.855), IgG levels after 5 (r=0.118, *P*=0.802), 10 (r=−0.401, *P*=0.372) and 15 (r=0.060, *P*=0.898) days, IgM levels after 5 (r=−0.025, *P*=0.958), 10 (r=−0.307, *P*=0.504) and 15 (r=−0.272, *P*=0.554) days of KLH injection, respectively (Table S1). If the data of the four/three seasons were pooled together, corticosterone levels were positively correlated with glucose levels (r=0.596, *P*<0.001), IgG levels after 10 (r=0.337, *P*=0.044) and 15 days (r=0.371, *P*=0.026) of KLH injection, leptin levels (r=0.651, *P*<0.001), but negatively with PHA response (r=−0.529, *P*<0.001) (Figs S1 and S2; Table S1). No correlations were observed between corticosterone levels and body mass (r=0.255, *P*=0.084), IgG levels post-5 days of KLH injection (r=0.075, *P*=0.665), IgM levels after 5 (r=0.227, *P*=0.184), 10 (r=0.270, *P*=0.111) and 15 (r=0.167, *P*=0.331) days of KLH injection (Tabel S1).

## DISCUSSION

In the present study, male striped hamsters demonstrated seasonal variations in body mass but not relative fatness. Many immunological parameters, including spleen mass, white blood cells, cellular immunity, IgG and IgM levels showed seasonal changes, whereas thymus mass had no seasonal changes. Seasonal variations were also observed in body fat mass, blood glucose and the levels of leptin and corticosterone in male hamsters.

Maximum body mass occurred in the summer in male hamsters, which was compatible with some animals including Brandt's voles (*Lasiopodomys brandtii*) (Li and Wang, 2005), root voles (*Microtus oeconomus*) (Wang et al., 2006a), plateau pikas (*Ochotona curzoniae*) (Wang et al., 2006b), meadow voles (*Microtus pennsylvanicus*) (Iverson and Turner, 1974) and Siberian hamsters (*Phodopus sungorus*) (Mercer, 1998), but incompatible with others including Golden hamsters (*Mesocricetus auratus*) (Steinlechner et al., 1983) and Collared lemmings (*Dicrostonys groenlandicus*) (Nagy et al., 1995), whose body masses were lower in summer than in other seasons. This result also disagreed with previous research in which body mass had no seasonal changes in female striped hamsters (Zhao et al., 2014). This discrepant result may be explained by the differences in sexes and animal origin – hamsters used in our study were captured wild, while hamsters in Zhao's study were from experimental environments and were acclimated to natural temperatures and photoperiods for 12 months (Zhao et al., 2014). No seasonal variation of the relative fatness was observed in male hamsters in the present study, inconsistent with previous research in which relative fatness was lower in summer but higher in the fall (Zhou et al., 1992). The differences in the geographic distribution and capture time might account for this discrepancy, in which female hamsters were captured in Huhehaote district from 1984 to 1989 in Zhou's research.

Immune organs such as the thymus and spleen are usually used to reflect immune function in field studies (Nelson and Demas, 1996; Calder and Kew, 2002; Smith and Hunt, 2004). The thymus is a central lymphoid organ in which bone marrow-derived T cell precursors undergo differentiation into macrophages, dendritic cells and so on, thus a larger thymus size indicates a stronger immune system (Savino and Dardenne, 2000). The spleen has many functions including lymphocyte production, antibody synthesis, erythrocyte storage and erythrolysis, hence a larger spleen is more effective at producing an immune response than a smaller one (Smith and Hunt, 2004). Thymus mass in our study had no seasonal changes, while spleen mass was higher in the fall than in other seasons in striped hamsters – the former does not, but the later does, support the winter immunoenhancement hypothesis (Sinclair and Lochmiller, 2000). The result of seasonal changes in spleen mass agreed with some species including the Northern red-backed mouse (*Clethrionomys rutilus*) (Sealander and Bickerstaff, 1967), the pine vole (*Microtus pinetorum*) (Valentine and Kirkpatrick, 1970) and the cotton rat (*Sigmodon hispidus*) (Lochmiller et al., 1994), but disagreed with others such as field voles (*Microtus agrestis*) (Newson, 1962) and Mongolian gerbils (*Meriones unguiculatus*) (Zhang and Wang, 2006) in which spleen mass was lower in winter than in other seasons. The reasons why seasonal shifts in spleen mass varied between hamsters and other species may be due to the differences in habitat settings, life history traits and so on. For example, the Mongolian gerbil is a social, diurnal species that inhabits mainly desert and semi-arid regions of northern China, which is completely different from the striped hamster in our present study (Zhang and Wang, 1998). Other immunological parameters besides immune organs are also important to indicate immune responses more completely, which include cellular and humoral immunity (Zhang and Wang, 2005; Calder and Kew, 2002).

Cellular immunity was higher in the fall and winter than in the summer in male hamsters, which was similar in Red deer (*Cervus elaphus*) (Fernández-de-Mera et al., 2011) and was in accordance with the winter immunoenhancement hypothesis (Sinclair and Lochmiller, 2000). Seasonally breeding small mammals including striped hamsters usually reproduce in the spring and summer, and most reproductive activities cease before winter begins, which are often accompanied by decreases in androgen secretion (Lu et al., 1987; Zhang and Wang, 1998; Martin et al., 2007). Generally, elevated levels of androgen associated with breeding activity contribute to the suppression of immune responses, and hence immune responses would increase in non-breeding seasons. Although we did not detect the concentration of testosterone in striped hamsters, the variation of testosterone might account for their higher cellular immunity in the fall and winter. Another reason of seasonal variations in immunity may be related with the changes in environmental signals such as temperature, food availability and photoperiod (Nelson and Demas, 1996). Cellular immunity in male striped hamsters was not influenced by cold stress and food restriction (Xu et al., 2017), implying that seasonal changes of cellular immunity in hamsters may be triggered by seasonal changes in the photoperiod. Animals in the non-tropical zone usually use photoperiods to anticipate seasonal changes, and immune responses in winter-like days are often higher than in summer-like days (Lochmiller, et al., 1994; Demas and Nelson, 1998; Brainard et al., 1987; Bilbo et al., 2002; Yellon et al., 1999; Martin et al., 2007; Stevenson and Prendergast, 2015). Enhancement of cellular immunity in the fall and winter might increase the resistance to intracellular infection (e.g. viruses) and hence increase the survival capacity in harsh winter in hamsters.

IgG and IgM titers also demonstrated seasonal variations in male hamsters. IgG levels after 10 and 15 days of KLH injection were higher in the summer than in the fall, and similarly IgM titers after 10 days of KLH injection were lowest in the fall among the three seasons (fall, spring and summer), which did not support the winter immunoenhancement hypothesis. These results agreed with research in ground squirrels (*Spermophilus beecheyi*) (Sidky et al., 1972), but disagreed with other species such as bank voles (*Clethrionomys glareolus*) (Saino et al., 2000) and Mongolian gerbils (*Meriones unguiculatus*) (Zhang and Wang, 2006). Lower humoral immunity suggested that the capacity of controlling extracellular pathogens was also lower in male hamsters in the fall compared with other seasons. Due to their usually solitary nature, during the breeding season the contact rates of male hamsters with their female mates or other males might increase, which would consequently increase the likelihood of infection. Moreover, higher pathogen pressures also usually occur in the breeding seasons, which may lead to higher bacterial, viral or parasitic infections. Therefore, hamsters enhanced their humoral immunity at these times to control the increased possibilities of infection and hence increase their survival capacity. The reasons why cellular immunity was enhanced in the fall, while humoral immunity was enhanced in spring and summer, may be related to the cost of different immunological components or different seasonal changes in distinct pathogens such as viruses, bacteria and parasites, which need further investigation in the future.

The changes in energy reserves and hormones profiles can usually underlie seasonal variations in immune responses. Energy resources such as body fat and blood glucose play a crucial role in expensive physiological processes such as immune responses (Demas et al., 1997; Moret and Schmid-Hempel, 2000; Martin et al., 2002; Demas, 2004; Trayhurn, 2005). Adipose tissues are considered immune and endocrine organs besides serving as energy reserves (Ahima and Flier, 2000; Matarese and La Cava, 2004; Trayhurn, 2005; Fantuzzi, 2005; Schäffler et al., 2007). In general, animals with low energy reserves allocate less energy to immune responses than those with higher reserves (Houston et al., 2007). Moreover, blood glucose is an important metabolic fuel which provides energy directly for mounting immune responses (Matarese and La Cava, 2004; Maciver et al., 2008; Xu and Wang, 2011). Consequently, reduction in body fat mass and glucose would harm immunity (Demas et al., 2003). From the fall of 2014 to the summer of 2015, both body fat mass and blood glucose increased with the seasons, which matched those of IgG and IgM titers, but was opposite to cellular immunity in male hamsters. Moreover, cellular immunity was negatively correlated with blood glucose but not with body fat mass, and no correlations were observed between body fat mass and IgG and IgM titers, implying that the changes in energy reserves could not explain seasonal changes in cellular and humoral immunity.

Leptin can regulate immune responses directly (Matarese et al., 2005; Lam and Lu, 2007; Steiner and Romanovsky, 2007), and lower leptin levels would impair immunity (Lord et al., 1998; Flier, 1998; Ahima and Flier, 2000). In the present study, leptin concentrations were higher in spring and summer than in fall and winter in male hamsters, which agreed with research in some species including Brandt's voles (*Microtus brandti*) (Li and Wang, 2005), Mongolian gerbils (*Meriones unguiculatus*) (Zhang and Wang, 2007), root voles (*Microtus oeconomus*) (Wang et al., 2006a), plateau pikas (*Ochotona curzoniae*) (Wang et al., 2006b), Chaotung voles (*Eothenomys olitor*) (Wan-Long and Zheng-Kun, 2015) and female striped hamsters (Zhao et al., 2014). However, this result was not consistent with the research in which leptin levels were highest in the winter and lowest in the summer in Microbiotherid marsupials (*Dromiciops gliroides*) (Franco et al., 2017). Leptin is a pleiotropic hormone and it also has a regulatory role in reproduction (Lam and Lu, 2007). The higher leptin levels in spring and summer in male hamsters might be related to the initiation of reproduction, which requires further investigation (Manfredi-Lozano et al., 2016). Leptin had no correlations with cellular immunity, IgG and IgM titers, implying that leptin did not mediate seasonal changes in cellular and humoral immunity.

Stress hormones such as corticosterone or cortisol often increase under stressful conditions, which usually have a suppressive effect on immune function (Sapolsky et al., 2000; Webster Marketon and Glaser, 2008). Seasonal variations in corticosterone or cortisol have also been examined in several wild animals (Romero et al., 2008; Vera et al., 2011). Corticosterone levels also showed seasonal changes in male hamsters and corticosterone levels were higher in spring and summer than in fall and winter in the present study. This result agreed with other findings such as striped mice (*Rhabdomys pumilio*) (Schradin, 2008) and degus (*Octodon degus*) (Quispe et al., 2014), in which corticosterone or cortisol levels were higher in the breeding season than in the non-breeding season. Increased corticosterone or cortisol levels during the breeding season implied that they might be more stressed due to social conflicts, or corticosterone might also help hamsters mobilize energy and hence cope with the increased energetic demands during this period (Quispe et al., 2014). Corticosterone levels were positively correlated with IgG titers after 10 and 15 days of KLH injection only in the fall, but were not correlated with IgG and IgM concentrations in spring and summer, respectively. No significant correlations were observed between corticosterone titers and cellular immunity in fall, winter, spring and summer. If the data of the three/four seasons were pooled together, corticosterone titers were negatively correlated with cellular immunity but positively correlated with IgG levels. These results implied that the role of corticosterone in humoral immunity varied during different seasons, and overall corticosterone had a suppressive effect on cellular immunity and enhancing effect on humoral immunity.

In summary, all immunological parameters except the thymus demonstrated seasonal variations, but their seasonal patterns were different in male striped hamsters. Spleen mass was highest in the fall, while white blood cells and cellular immunity were lowest in the summer among the four seasons, which supports the winter immunoenhancement hypothesis. IgG and IgM titers were lowest in the fall, which is against this hypothesis. Body fat mass had no correlations with cellular and humoral immunity, suggesting it was not the reason for seasonal changes in cellular and humoral immunity in males. Hormone profiles including leptin and corticosterone exhibited similar seasonal changes in which their titers were higher in spring and summer than in fall and winter. The results suggested that the former did not mediate seasonal changes in cellular and humoral immunity, while the latter might have boosting effects on humoral immunity but suppressive effects on cellular immunity. Whether other hormones such as melatonin and testosterone would mediate seasonal variations in immune responses deserves further research (Carrillo-Vico et al., 2005; Martin et al., 2007; Weil et al., 2015).

## MATERIALS AND METHODS

### Animals and experimental design

All animal procedures were carried out according to the guidelines of the Animal Care and Use Committee of Qufu Normal University. Adult male striped hamsters used in this study were captured from Jiuxian Mountain (35°46.275′N, 116°59.976′E) in Qufu of the Shangdong province, China. Fifteen male hamsters were captured during November 2014 (the fall group, mean temperature was 9.3°C); 11 males were captured during January 2015 (the winter group, mean temperature was 2.9°C); 14 males were captured during March and April 2015 (the spring group, mean temperature was 13.1°C); and eight males were captured during June 2015 (the summer group, mean temperature was 25.9°C). After the hamsters were carried to experimental room in Qufu Normal University (35.39′N, 116.98′E), their body mass (W; g) and body length (L; cm) were measured to calculate the relative fatness (K) (K=100 W/L^3^) (Zhou et al., 1992). These hamsters were housed individually in plastic cages (30 cm×15 cm×20 cm) with sawdust as bedding under semi-natural conditions (inside the pavilion). Standard rat pellet chow (Beijing KeAo Feed Co., Beijing, China) and water were provided *ad libitum* throughout of the experiment. The macronutrient composition of the diet was 6.2% crude fat, 18% crude protein, 23.1% neutral fiber, 5% crude fiber, 12.5% acid detergent fiber and 10.0% ash and the caloric value was 17.5 kJ/g. After about 2 days, their PHA responses were examined. Then, hamsters were injected with KLH (Sigma-Aldrich, LH7017) to assess humoral immunity (the detailed procedures are described below).

### Body composition

Body composition was measured according to Xu and Wang (2010). In brief, immune organs including thymus and spleen were dissected and weighed (±1 mg). All the visceral organs were removed to obtain wet carcass. Moreover, subcutaneous fat, retroperitoneal fat, perigonadal fat and mesenteric fat were also dissected carefully and weighed. All four parts of fat mass together were regarded as total body fat mass. The percent content of subcutaneous fat, retroperitoneal fat, perigonadal fat, mesenteric fat and total body fat mass was divided by the mass of wet carcass, respectively (Xu et al., 2017).

### White blood cells assays

At the end of the experiment, after collecting trunk blood, 20 µl whole blood was diluted immediately in 4 ml diluent and white blood cells were counted in the Hematology Analyzer (Auto Counter 910EO^+^) (Xu et al., 2017).

### Cellular immunity assays

PHA response was measured as described previously (Goüy de Bellocq et al., 2006; Xu and Wang, 2011). Specifically, hamsters were caught carefully, then we measured the footpad thickness of the left hind foot with a micrometer (Digimatic Indicator ID-C Mitutoyo Absolute cod. 547-301, Japan) to ±0.01 mm. Immediately thereafter, hamsters were injected subcutaneously 0.1 mg of PHA (PHA-P, Sigma-Aldrich, L-8754) dissolved in 0.03 ml of sterile saline (pH7.4) in the middle of the footpad. After 6 h, 12 h, 24 h, 48 h and 72 h injection, we measured the footpad thickness. The PHA response (i.e. cellular immunity) was calculated as the difference between pre- and post-injection measurements divided by the initial footpad thickness [PHA response=(post PHA−pre PHA)/pre PHA]. Six measures of footpad thickness were taken to obtain the value of each hamster (Xu and Hu, 2017). Only the 6 h data were included in the results because they were representative of the maximal response.

### Humoral immunity assays

After measuring PHA responses, hamsters in the four seasons received a single subcutaneous injection of 100 μg of KLH (Sigma-Aldrich, LH7017) suspended in 0.1 ml sterile saline in order to assess humoral immunity. After 5 and 10 days of KLH injection, hamsters in all the groups were lightly anesthetized with isoflurane (Shandong LiNuo Pharmaceutical Co.) and blood samples (∼300 μl) were drawn from the retro-orbital sinus for later measurement of anti-KLH IgM and IgG concentrations. After another 5 days (i.e. after 15 days of KLH injection), each hamster was euthanized and trunk blood was collected for measurements of anti-KLH IgM and IgG, white blood cells, glucose, leptin and corticosterone. IgM is the first immunoglobulin class and IgG is the predominant immunoglobulin class present in the blood produced following an immune challenge (Demas et al., 2003; Zysling and Demas, 2007). Blood samples were allowed to clot for 1 h and the samples were centrifuged at 4°C for 30 min at 4000 rpm. Sera were collected and stored in polypropylene microcentrifuge tubes at −20°C until assayed.

Enzyme-linked immunosorbent assay (ELISA) was used to assess serum IgM and IgG concentrations (Demas et al., 2003; Zysling and Demas, 2007; Xu et al., 2017). Specifically, microtiter plates were coated with 100 μl 0.5 mg/ml KLH in sodium bicarbonate buffer (pH 9.6) overnight at 4°C. Plates were washed with 200 μl phosphate buffered saline containing 0.05% Tween 20 (PBS-T, pH 7.4) three times, then blocked with 5% non-fat dry milk in PBS-T overnight at 4°C to reduce non-specific binding, and washed again with PBS-T three times. Thawed serum samples were diluted 1:20 with PBS-T, and 150 µl of each serum dilution was added in duplicate to the wells of the antigen-coated plates. Positive control samples (pooled sera from KLH repeatedly injected hamsters, similarly diluted with PBS-T) and negative control samples (pooled sera from KLH-naïve hamsters, similarly diluted with PBS-T) were added in duplicate. Plates were sealed, incubated at 37°C for 3 h, and then washed with PBS-T three times. Secondary antibody (alkaline phosphatase-conjugated-anti mouse IgG diluted 1:2000 with PBS-T, Sigma-Aldrich; alkaline phosphatase-conjugated-anti mouse IgM diluted 1:500 with PBS-T, Sigma-Aldrich) was added to the wells, and the plates were sealed and incubated for 1 h at 37°C. Plates were then washed again with PBS-T and 150 μl enzyme substrate p-nitrophenyl phosphate (1 mg/ml in diethanolamine substrate buffer; Sigma-Aldrich) was added to each well. Plates were protected from light during the enzyme-substrate reaction, which was terminated after 30 min by adding 50 µl of 1.5 mol/l NaOH solution to each well. The optical density (OD) of each well was determined using a plate reader (Bio-Rad) equipped with a 405 nm wavelength filter, and the mean OD for each set of duplicate wells was calculated. To minimize inter- and intra-assay variability, the mean OD for each sample was expressed as a ratio of its plate positive control OD for statistical analysis (Demas et al., 2003; Zysling and Demas, 2007). The blood sample in the winter was insufficient for assessing the titers of anti-KLH IgG and IgM, so the data of IgG and IgM titers in the winter was lacking.

### Blood glucose assays

Blood glucose concentrations were measured with FreeStyle Mini Blood Meter (Abbott Diabetes Care Inc., Alameda, USA) according to the manufacturer’s instructions. The range of blood glucose tested was 20–550 mg/dl. The within-lot and -vial precision are <5.6% and <4.1%, respectively.

### Serum leptin assays

Serum leptin concentrations were determined by hamster leptin ELISA kit (Cat. no. XL-85K, Linco Research Inc., Missouri, USA). The range detected by this assay was 0.3–8 ng/ml when using a 10 µl sample (see manufacturer's instructions for hamster leptin ELISA Kit). The detailed procedure was carried out as per the manufacturer's instructions of the hamster leptin ELISA kit.

### Serum corticosterone assays

Serum corticosterone (CORT) concentrations were determined by hamster corticosterone ELISA kit (Cat. no. HR083, RapidBio Lab. Calabasas, California, USA). The range detected by this assay was 8–150 ng/ml when using a 10 µl sample. The detailed procedure followed the manufacturer's instructions of the hamster corticosterone ELISA kit.

### Statistical analysis

Data were analyzed using SPSS 18.0 software (SPSS Inc., Chicago, USA). Prior to all statistical analyses, data were examined for normality and homogeneity of variance, using Kolmogorov–Smirnov and Levene tests, respectively. The ratio values of PHA response were subjected to arcsine transformation. Group differences in thymus and spleen mass with final body mass as the covariate were analyzed by General Linear Model multivariate analysis followed by Bonferroni *post hoc* tests. Group differences in other parameters (body compositions, white blood cells, PHA response, IgM and IgG concentrations, blood glucose, leptin and corticosterone) were analyzed by one-way ANOVA followed by Tukey's *post hoc* tests. Pearson correlation analysis was performed to determine the correlations of PHA response, IgM and IgG titers with body fat mass, blood glucose, leptin and corticosterone concentrations for all hamsters. Results are presented as mean±s.e., and *P*<0.05 was considered to be statistically significant.

## Supplementary Material

Supplementary information

## References

[BIO038489C1] Ahima R. S. and Flier J. S. (2000). Adipose tissue as an endocrine organ. *Trends Endocrinol. Metab.* 11, 327-332. 10.1016/S1043-2760(00)00301-5 10996528

[BIO038489C2] Bilbo S. D., Dhabhar F. S., Viswanathan K., Saul A., Yellon S. M. and Nelson R. J. (2002). Short day lengths augment stress-induced leukocyte trafficking and stress-induced enhancement of skin immune function. *Proc. Natl. Acad. Sci. USA* 99, 4067-4072. 10.1073/pnas.062001899 11904451 PMC122649

[BIO038489C3] Brainard G. C., Knobler R. L., Podolin P. L., Lavasa M. and Lubin F. D. (1987). Neuroimmunology: modulation of the hamster immune system by photoperiod. *Life Sci.* 40, 1319-1326. 10.1016/0024-3205(87)90589-3 3561152

[BIO038489C4] Calder P. C. and Kew S. (2002). The immune system: a target for functional foods? *Br. J. Nutr.* 88, S165-S176. 10.1079/BJN2002682 12495459

[BIO038489C5] Carrillo-Vico A., Guerrero J. M., Lardone P. J. and Reiter R. J. (2005). A review of the multiple actions of melatonin on the immune system. *Endocrine* 27, 189-200. 10.1385/ENDO:27:2:189 16217132

[BIO038489C6] Demas G. E. (2004). The energetics of immunity: a neuroendocrine link between energy balance and immune function. *Horm. Behav.* 45, 173-180. 10.1016/j.yhbeh.2003.11.002 15047012

[BIO038489C7] Demas G. E. and Nelson R. J. (1998). Short-day enhancement of immune function is independent of steroid hormones in deer mice (*Peromyscus maniculatus*). *J. Comp. Physiol. B* 168, 419-426. 10.1007/s003600050161 9747522

[BIO038489C8] Demas G. E., Chefer V., Talan M. I. and Nelson R. J. (1997). Metabolic costs of mounting an antigen-stimulated immune response in adult and aged C57BL/6J mice. *Am. J. Physiol. Reg. Integr. Comp. Physiol.* 273, R1631-R1637. 10.1152/ajpregu.1997.273.5.R1631 9374803

[BIO038489C9] Demas G. E., Drazen D. L. and Nelson R. J. (2003). Reductions in total body fat decrease humoral immunity. *Proc. R. Soc. B* 270, 905-911. 10.1098/rspb.2003.2341 PMC1691330 12803904

[BIO038489C10] Dobrowolska A. and Adamczewska-Andrzejewska K. A. (1991). Seasonal and long-term changes in serum gamma-globulin levels in comparing the physiology and population density of the common vole, *Microtus arvalis Pall*. 1779. *J. Interdis. Cycle Res.* 22, 1-19. 10.1080/09291019109360094

[BIO038489C11] Dobrowolska A., Rewkiewicz-Dziarska A., Szarska I. and Gill J. (1974). Seasonal changes in haematological parameters, level of serum proteins and glycoproteins, activity of the thyroid gland, suprarenals and kidneys in the common vole (*Microtus arvalis Pall.*). *J. Interdis. Cycle Res.* 5, 347-354. 10.1080/09291017409359449

[BIO038489C12] Drazen D. L., Kriegsfeld L. J., Schneider J. E. and Nelson R. J. (2000). Leptin, but not immune function, is linked to reproductive responsiveness to photoperiod. *Am. J. Physiol. Regul. Integr. Comp. Physiol.* 278, R1401-R1407. 10.1152/ajpregu.2000.278.6.R1401 10848504

[BIO038489C13] Drazen D. L., Jasnow A. M., Nelson R. J. and Demas G. E. (2002). Exposure to short days, but not short-term melatonin, enhances humoral immunity of male Syrian hamsters (*Mesocricetus auratus*). *J. Pineal Res.* 33, 118-124. 10.1034/j.1600-079X.2002.02915.x 12153446

[BIO038489C14] Fantuzzi G. (2005). Adipose tissue, adipokines, and inflammation. *J. Allergy Clin. Immunol.* 115, 911-919. 10.1016/j.jaci.2005.02.023 15867843

[BIO038489C15] Fantuzzi G. and Faggioni R. (2000). Leptin in the regulation of immunity, inflammation, and hematopoiesis. *J. Leuk. Biol.* 68, 437-446. 11037963

[BIO038489C16] Fernández-de-Mera I. G., Jaroso R., Martín-Hernando M. P., Queiros J., Carta T., Ortiz J. A., Vicente J. and Gortázar C. (2011). The testing season affects red deer skinfold increase in response to phytohaemagglutinin. *Prevent. Veterin. Med.* 100, 79-83. 10.1016/j.prevetmed.2011.02.011 21440317

[BIO038489C17] Flier J. S. (1998). Lowered leptin slims immune response. *Nat. Med.* 4, 1124-1125. 10.1038/2619 9771741

[BIO038489C18] Franco M., Contreras C., Place N. J., Bozinovic F. and Nespolo R. F. (2017). Leptin levels, seasonality and thermal acclimation in the Microbiotherid marsupial *Dromiciops gliroides*: does photoperiod play a role? *Comp. Biochem. Physiol. A* 203, 233-240. 10.1016/j.cbpa.2016.09.025 27705753

[BIO038489C19] Goüy de Bellocq J. G., Krasnov B. R., Khokhlova I. S. and Pinshow B. (2006). Temporal dynamics of a T-cell mediated immune response in desert rodents. *Comp. Biochem. Physiol. A* 145, 554-559. 10.1016/j.cbpa.2006.08.045 17052931

[BIO038489C20] Houston A. I., McNamara J. M., Barta Z. and Klasing K. C. (2007). The effect of energy reserves and food availability on optimal immune defence. *Proc. R. Soc. B* 274, 2835-2842. 10.1098/rspb.2007.0934 PMC2373797 17848371

[BIO038489C21] Iverson S. L. and Turner B. N. (1974). Winter weight dynamics in Microtus pennsylvanicus. *Ecology* 55, 1030-1041. 10.2307/1940353

[BIO038489C23] Lago F., Dieguez C., Gómez-Reino J. and Gualillo O. (2007). Adipokines as emerging mediators of immune response and inflammation. *Nat. Clin. Pract. Rheum.* 3, 716-724. 10.1038/ncprheum0674 18037931

[BIO038489C24] Lam Q. L. K. and Lu L. W. (2007). Role of leptin in immunity. *Cell. Mol. Immunol.* 4, 1-13. 17349207

[BIO038489C25] Li X. S. and Wang D. H. (2005). Regulation of body weight and thermogenesis in seasonally acclimatized Brandt's voles (*Microtus brandti*). *Horm. Behav.* 48, 321-328. 10.1016/j.yhbeh.2005.04.004 15935352

[BIO038489C26] Lochmiller R. L., Vesty M. R. and McMurray S. T. (1994). Temporal variation in humoral and cell-mediated immune response in a *Sigmodon hispidus* population. *Ecology* 75, 236-245. 10.2307/1939397

[BIO038489C27] Lord G. M., Matarese G., Howard J. K., Baker R. J., Bloom S. R. and Lechler R. I. (1998). Leptin modulates the T-cell immune response and reverses starvation-induced immunosuppression. *Nature* 394, 897-901. 10.1038/29795 9732873

[BIO038489C28] Lu H. Q., Li Y. C. and Zhang X. D. (1987). Age determination, age structure and population dynamics of striped hamster. *Acta Theriol. Sin.* 7, 28-34. (in Chinese with English subtract).

[BIO038489C29] Maciver N. J., Jacobs S. R., Wieman H. L., Wofford J. A., Coloff J. L. and Rathmell J. C. (2008). Glucose metabolism in lymphocytes is a regulated process with significant effects on immune cell function and survival. *J. Leuk. Biol.* 84, 949-957. 10.1189/jlb.0108024 PMC2638731 18577716

[BIO038489C30] Manfredi-Lozano M., Roa J., Ruiz-Pino F., Piet R., Garcia-Galiano D., Pineda R., Zamora A., Leon S., Sanchez-Garrido M. A., Romero-Ruiz A. et al. (2016). Defining a novel leptin-melanocortin-kisspeptin pathway involved in the metabolic control of puberty. *Mol. Metab.* 5, 844-857. 10.1016/j.molmet.2016.08.003 27688998 PMC5034608

[BIO038489C31] Mann D. R., Akinbami M. A., Gould K. G. and Ansari A. A. (2000). Seasonal variations in cytokine expression and cell-mediated immunity in male rhesus monkeys. *Cell. Immunol.* 200, 105-115. 10.1006/cimm.2000.1623 10753502

[BIO038489C32] Martin L. B., Scheuerlein A. and Wikelski M. (2002). Immune activity elevates energy expenditure of house sparrows: a link between direct and indirect costs? *Proc. R. Soc. B* 270, 153-158. 10.1098/rspb.2002.2185 PMC1691219 12590753

[BIO038489C33] Martin L. B., Weil Z. M. and Nelson R. J. (2007). Seasonal changes in vertebrate immune activity: mediation by physiological trade-offs. *Philos. Trans R. Soc. B* 2142, 1-19. 10.1098/rstb.2007.2142 PMC2606753 17638690

[BIO038489C34] Matarese G. and La Cava A. (2004). The intricate interface between immune system and metabolism. *Trends Immunol.* 25, 193-200. 10.1016/j.it.2004.02.009 15039046

[BIO038489C35] Matarese G., Moschos S. and Mantzoros C. S. (2005). Leptin in immunology. *J. Immunol.* 174, 3137-3142. 10.4049/jimmunol.174.6.3137 15749839

[BIO038489C36] Mercer J. G. (1998). Regulation of appetite and body weight in seasonal Mammals. *Comp. Biochem. Physiol.* 119, C295-C303. 10.1016/S0742-8413(98)00018-8 9827002

[BIO038489C37] Moret Y. and Schmid-Hempel P. (2000). Survival for immunity: the price of immune system activation for bumblebee workers. *Science* 290, 1166-1168. 10.1126/science.290.5494.1166 11073456

[BIO038489C38] Moshkin M. P., Dobrotvorsky A. K., Mak V. V., Panov V. V. and Dobrotvorskaya E. A. (1998). Variability of immune response to heterologous erythrocytes during population cycles of red (*Clethrionomys rutilus*) and bank (*C. glareolus*) voles. *Oikos* 82, 131-138. 10.2307/3546923

[BIO038489C39] Nagy T. R., Gower B. A. and Stetson M. H. (1995). Endocrine correlates of seasonal body mass dynamics in the collared lemming (*Dicrostonyx groenlandicus*). *Am. Zool.* 35, 246-258. 10.1093/icb/35.3.246

[BIO038489C40] Nelson R. J. (2004). Seasonal immune function and sickness responses. *Trends Immunol.* 25, 187-192. 10.1016/j.it.2004.02.001 15039045

[BIO038489C41] Nelson R. J. and Demas G. E. (1996). Seasonal changes in immune function. *Q. Rev. Biol.* 71, 511-548. 10.1086/419555 8987173

[BIO038489C42] Nelson R. J., Demas G. E., Klein S. L. and Kriegsfeld L. J. (2002). *Seasonal Patterns of Stress, Immune Function, and Disease*. New York, NY: Cambridge University Press.

[BIO038489C43] Newson J. (1962). Seasonal differences in reticulocyte count, haemoglobin level and spleen weight in wild voles. *Br. J. Haematol.* 8, 296-302. 10.1111/j.1365-2141.1962.tb06524.x 14479354

[BIO038489C44] Owens I. P. F. and Wilson K. (1999). Immunocompetence: a neglected life history trait or conspicuous red herring? *Trends Ecol. Evol.* 14, 170-172. 10.1016/S0169-5347(98)01580-8

[BIO038489C45] Pond C. M. (1996). Interactions between adipose tissue and the immune system. *Proc. Nutr. Soc.* 55, 111-126. 10.1079/PNS19960014 8832785

[BIO038489C46] Quispe R., Villavicencio C. P., Addis E., Wingfield J. C. and Vasquez R. A. (2014). Seasonal variations of basal cortisol and high stress response to captivity in *Octodon degus*, a mammalian model species. *Gen. Comp. Endocr.* 197, 65-72. 10.1016/j.ygcen.2013.12.007 24368258

[BIO038489C47] Romero L. M., Meister C. J., Cyr N. E., Kenagy G. J. and Wingfield J. C. (2008). Seasonal glucocorticoid responses to capture in wild free-living mammals. *Am. J. Physiol.Reg. Integr. Comp. Physiol.* 294, R614-R622. 10.1152/ajpregu.00752.2007 18094060

[BIO038489C48] Saino N., Canova L., Fasola M. and Martinelli R. (2000). Reproduction and population density affect humoral immunity in bank voles under field experimental conditions. *Oecologia* 124, 358-366. 10.1007/s004420000395 28308773

[BIO038489C49] Sapolsky R. M., Romero L. M. and Munck A. U. (2000). How do glucocorticoids influence stress responses? Integrating permissive, suppressive, stimulatory, and preparative actions. *Endocr. Rev.* 21, 55-89. 10.1210/er.21.1.55 10696570

[BIO038489C50] Savino W. and Dardenne M. (2000). Neuroendocrine control of thymus physiology. *Endocr. Rev.* 21, 412-443. 10.1210/er.21.4.412 10950159

[BIO038489C51] Schäffler A., Schölmerich J. and Salzberger B. (2007). Adipose tissue as an immunological organ: toll-like receptors, C1q/TNFs and CTRPs. *Trends Immunol.* 28, 393-399. 10.1016/j.it.2007.07.003 17681884

[BIO038489C52] Schradin C. (2008). Seasonal changes in testosterone and corticosterone levels in four social classes of a desert dwelling sociable rodent. *Horm. Behav.* 53, 573-579. 10.1016/j.yhbeh.2008.01.003 18279873

[BIO038489C53] Sealander J. A. and Bickerstaff L. K. (1967). Seasonal changes in reticulocyte number and in relative weights of the spleen, thymus, and kidneys in the Northern red-backed mouse. *Can. J. Zool.* 45, 253-260. 10.1139/z67-036 6043727

[BIO038489C54] Sheldon B. C. and Verhulst S. (1996). Ecological immunology: costly parasite defences and trade-offs in evolutionary ecology. *Trends Ecol. Evol.* 11, 317-321. 10.1016/0169-5347(96)10039-2 21237861

[BIO038489C55] Sidky Y. A., Hayward J. S. and Ruth R. F. (1972). Seasonal variations of the immune response of ground squirrels kept at 22–24°C. *Can. J. Physiol. Pharmacol.* 50, 203-206. 10.1139/y72-031 4555721

[BIO038489C56] Sinclair J. A. and Lochmiller R. L. (2000). The winter immunoenhancement hypothesis: associations among immunity, density, and survival in Prairie vole (*Microtus ochrogaster*) populations. *Can. J. Zool.* 78, 254-264. 10.1139/z99-203

[BIO038489C57] Smith K. G. and Hunt J. L. (2004). On the use of spleen mass as a measure of avian immune system strength. *Oecologia* 138, 28-31. 10.1007/s00442-003-1409-y 14576931

[BIO038489C58] Smits J. E., Bortolotti G. R. and Tella J. L. (1999). Simplifying the phytohaemagglutinin skin-testing technique in studies of avian immunocompetence. *Funct. Ecol.* 13, 567-572. 10.1046/j.1365-2435.1999.00338.x

[BIO038489C59] Song Z.-G. and Wang D.-H. (2003). Metabolism and thermoregulation in the striped hamster *Cricetulus barabensis*. *J. Therm. Biol.* 28, 509-514. 10.1016/S0306-4565(03)00051-2

[BIO038489C60] Steiner A. A. and Romanovsky A. A. (2007). Leptin: at the crossroads of energy balance and systemic inflammation. *Prog. Lipid Res.* 46, 89-107. 10.1016/j.plipres.2006.11.001 17275915 PMC1976277

[BIO038489C61] Steinlechner S., Heldmaier G. and Becker H. (1983). The seasonal cycle of body weight in the Djungarian hamster: photoperiodic control and the influence of starvation and melatonin. *Oecologia* 60, 401-405. 10.1007/BF00376859 28310703

[BIO038489C62] Stevenson T. J. and Prendergast B. J. (2015). Photoperiodic time measurement and seasonal immunological plasticity. *Front. Neuroendocr.* 37, 76-88. 10.1016/j.yfrne.2014.10.002 PMC4405432 25456046

[BIO038489C63] Trayhurn P. (2005). Endocrine and signalling role of adipose tissue: new perspectives on fat. *Acta. Physiol. Scand.* 184, 285-293. 16026420 10.1111/j.1365-201X.2005.01468.x

[BIO038489C64] Valentine G. L. and Kirkpatrick R. L. (1970). Seasonal changes in reproductive and related organs in the pine vole, *Microtus pinetorum*, in south-western Virginia. *J. Mammal.* 51, 553-560. 10.2307/1378394 5483408

[BIO038489C65] Vera F., Antenucci C. D. and Zenuto R. R. (2011). Cortisol and corticosterone exhibit different seasonal variation and responses to acute stress and captivity in tucotucos (*Ctenomys talarum*). *Gen. Comp. Endocr.* 170, 550-557. 10.1016/j.ygcen.2010.11.012 21095193

[BIO038489C66] Wan-Long Z. and Zheng-Kun W. (2015). Seasonal changes in body mass, serum leptin levels and hypothalamic neuropeptide gene expression in male *Eothenomys olitor*. *Comp. Biochem. Physiol. A* 184, 83-89. 10.1016/j.cbpa.2015.02.011 25700741

[BIO038489C67] Wang J.-M., Zhang Y.-M. and Wang D.-H. (2006a). Seasonal regulations of energetics, serum concentrations of leptin, and uncoupling protein 1 content of brown adipose tissue in root voles (*Microtus oeconomus*) from the Qinghai-Tibetan plateau. *J. Comp. Physiol.* 176, B663-B671. 10.1007/s00360-006-0089-4 16786335

[BIO038489C68] Wang J.-M., Zhang Y.-M. and Wang D.-H. (2006b). Seasonal thermogenesis and body mass regulation in plateau pikas (*Ochotona curzoniae*). *Oecologia* 149, 373-382. 10.1007/s00442-006-0469-1 16823564

[BIO038489C69] Webster Marketon J. I. W. and Glaser R. (2008). Stress hormones and immune function. *Cell. Immunol.* 252, 16-26. 10.1016/j.cellimm.2007.09.006 18279846

[BIO038489C70] Weil Z. M., Borniger J. C., Cisse Y. M., Abi Salloum B. A. and Nelson R. J. (2015). Neuroendocrine control of photoperiodic changes in immune function. *Front. Neuroendocr.* 37, 108-118. 10.1016/j.yfrne.2014.10.001 PMC4402123 25456047

[BIO038489C71] Xu D.-L. and Hu X.-K. (2017). Photoperiod and temperature differently affect immune function in striped hamsters (*Cricetulus barabensis*). *Comp. Biochem. Physiol. A* 204, 211-218. 10.1016/j.cbpa.2016.12.009 27956167

[BIO038489C72] Xu D.-L. and Wang D.-H. (2010). Fasting suppresses T cell-mediated immunity in female Mongolian gerbils (*Meriones unguiculatus*). *Comp. Biochem. Physiol. A* 155, 25-33. 10.1016/j.cbpa.2009.09.003 19748595

[BIO038489C73] Xu D.-L. and Wang D.-H. (2011). Glucose supplement reverses the fasting-induced suppression of cellular immunity in Mongolian gerbils (Meriones unguiculatus). *Zoology* 114, 306-312. 10.1016/j.zool.2011.04.002 21885265

[BIO038489C75] Xu D.-L., Hu X.-K. and Tian Y.-F. (2017). Effect of temperature and food restriction on immune function in striped hamsters (*Cricetulus barabensis*). *J. Exp. Biol.* 220, 2187-2195. 10.1242/jeb.153601 28381582

[BIO038489C76] Yellon S. M., Teasley L. A., Fagoaga O. R., Nguyen H. C., Truong H. N. and Nehlsen-Cannarella S. L. (1999). Role of photoperiod and the pineal gland in T cell-dependent humoral immune reactivity in the Siberian hamster. *J. Pineal Res.* 27, 243-248. 10.1111/j.1600-079X.1999.tb00622.x 10551773

[BIO038489C77] Zhang Z. B. and Wang Z. W. (1998). *Ecology and Management of Rodent Pests in Agriculture*. Beijing: Ocean Publishing House.

[BIO038489C78] Zhang Z. Q. and Wang D. H. (2005). Animal immunocompetence and its effect on population regulation and life history trade-off. *Chin. J. Appl. Ecol.* 16, 1375-1379. (in Chinese). 16252887

[BIO038489C79] Zhang Z. Q. and Wang D. H. (2006). Seasonal changes in immune function, body fat mass and organ mass in Mongolian gerbils (*Meriones unguiculatus*). *Acta Theriol. Sin.* 26, 338-345. (In Chinese with English summary).

[BIO038489C80] Zhang Z.-Q. and Wang D.-H. (2007). Seasonal changes in thermogenesis and body mass in wild Mongolian gerbils (*Meriones unguiculatus*). *Comp. Biochem. Physiol. A* 148, 346-353. 10.1016/j.cbpa.2007.05.012 17588796

[BIO038489C81] Zhang Y., Proenca R., Maffei M., Barone M., Leopold L. and Friedman J. M. (1994). Positional cloning of the mouse obese gene and its human homologue. *Nature* 372, 425-432. 10.1038/372425a0 7984236

[BIO038489C82] Zhao Z.-J., Cao J., Meng X.-L. and Li Y.-B. (2010). Seasonal variations in metabolism and thermoregulation in the striped hamster (*Cricetulus barabensis*). *J. Therm. Biol.* 35, 52-57. 10.1016/j.jtherbio.2009.10.008

[BIO038489C83] Zhao Z. J., Cao J. and Chen K. X. (2014). Seasonal changes in body mass and energy budget in striped hamsters. *Acta Theriol. Sin.* 34, 149-157. 10.1086/674974 24642542

[BIO038489C84] Zhou Y. L., Hou X. X., Dong W. H. and Yang Y. P. (1992). A study of the relative fatness of striped hamster. *Acta Theriol. Sin.* 12, 207-212.

[BIO038489C85] Zysling D. A. and Demas G. E. (2007). Metabolic stress suppresses humoral immune function in long-day, but not short-day, Siberian hamsters (*Phodopus sungorus*). *J. Comp. Physiol. B* 177, 339-347. 10.1007/s00360-006-0133-4 17149587

[BIO038489C86] Zysling D. A., Garst A. D. and Demas G. E. (2009). Photoperiod and food restriction differentially affect reproductive and immune responses in Siberian hamsters *Phodopus sungorus*. *Funct. Ecol.* 23, 979-988. 10.1111/j.1365-2435.2009.01572.x

