# Supplementary material for: Seasonal variations in cellular and humoral immunity in male striped hamsters (Cricetulus barabensis)
Source: Biol Open. 2018 Nov 7;7(12):bio038489. doi: 10.1242/bio.038489 (PMC6310883; doi:10.1242/bio.038489)
Supplement: Supplementary information [file biolopen-7-038489-s1.pdf]

Biology Open (2018): doi:10.1242/bio.038489: Supplementary information

Fig. S1. The correlations between corticosterone and IgG titres after 10 (A), 15 (B) after KLH injection in the fall, IgG titres after 10 (C), 15 (D) after KLH injection in the three seasons.

A

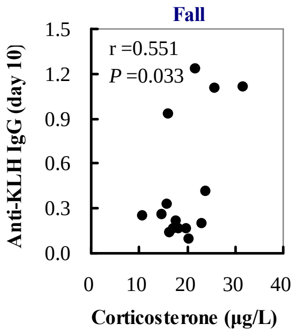

B

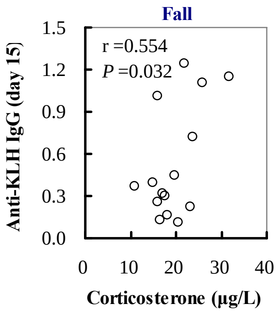

C

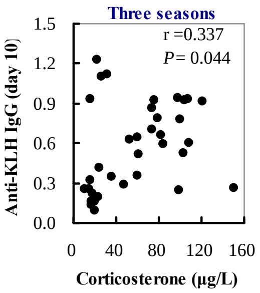

D

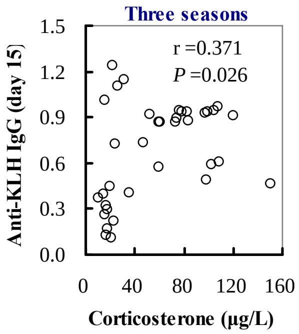

Biology Open • Supplementary information

Fig. S2. The correlations between PHA response and body fat mass (A), blood glucose (B) and corticosterone (C) in the four seasons.

A

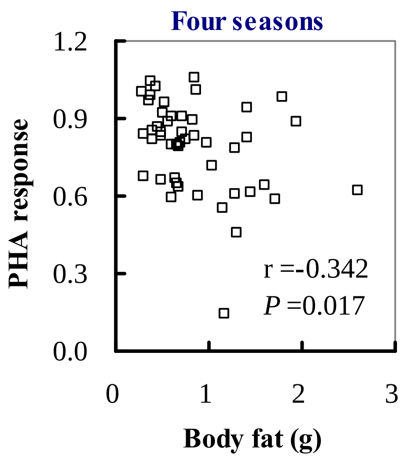

B

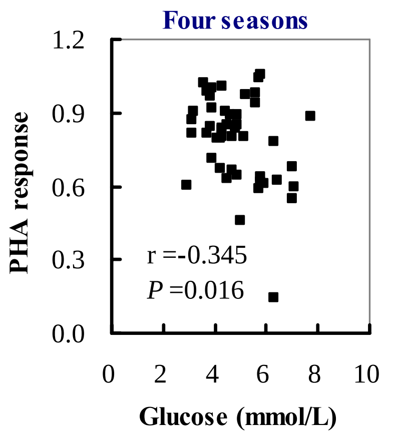

C

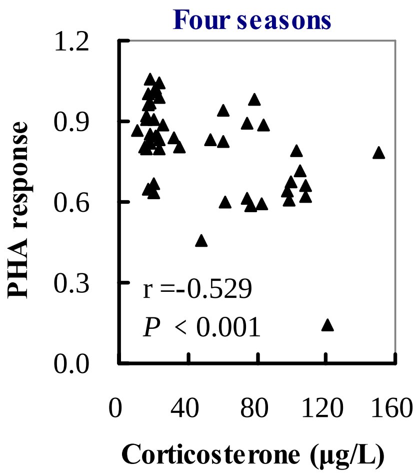

Biology Open • Supplementary information

Table S1. The correlations between energy status, hormone profiles and PHA response, humoral immunity in striped male hamsters

		PHA		IgG 5		IgG 10		IgG 15		IgM 5		IgM 10		IgM 15	
		r	*P*	r	*P*	r	*P*	r	*P*	r	*P*	r	*P*	r	*P*
Fall (n=15)	Body fat	-0.207	ns	-0.106	ns	-0.166	ns	-0.213	ns	0.110	ns	0.120	ns	0.003	ns
	Glucose	0.130	ns	0.270	ns	0.296	ns	0.164	ns	-0.294	ns	-0.221	ns	-0.309	ns
	Leptin	0.186	ns	-0.306	ns	-0.272	ns	-0.320	ns	0.265	ns	0.127	ns	0.249	ns
	CORT	0.047	ns	0.459	ns	0.551	0.033	0.554	0.032	-0.483	ns	-0.490	ns	-0.363	ns
Winter (n=11)	Body fat	0.437	ns	-	-	-	-	-	-	-	-	-	-	-	-
	Glucose	0.069	ns	-	-	-	-	-	-	-	-	-	-	-	-
	Leptin	-0.455	ns	-	-	-	-	-	-	-	-	-	-	-	-
	CORT	0.024	ns	-	-	-	-	-	-	-	-	-	-	-	-
Spring (n=14)	Body fat	0.288	ns	-0.005	ns	-0.468	ns	-0.394	ns	-0.208	ns	-0.282	ns	0.218	ns
	Glucose	0.226	ns	0.053	ns	-0.044	ns	-0.067	ns	-0.178	ns	-0.273	ns	0.002	ns
	Leptin	0.298	ns	0.466	ns	-0.078	ns	0.108	ns	0.124	ns	-0.059	ns	0.267	ns
	CORT	-0.024	ns	-0.010	ns	0.066	ns	-0.116	ns	-0.180	ns	-0.435	ns	0.102	ns
Summer (n=8)	Body fat	-0.132	ns	-0.518	ns	0.102	ns	-0.591	ns	-0.449	ns	-0.248	ns	-0.185	ns
	Glucose	-0.305	ns	-0.604	ns	-0.684	ns	-0.329	ns	-0.442	ns	-0.581	ns	-0.617	ns
	Leptin	-0.546	ns	-0.464	ns	-0.003	ns	-0.344	ns	0.076	ns	-0.086	ns	-0.020	ns
	CORT	0.086	ns	0.118	ns	-0.401	ns	0.060	ns	-0.025	ns	-0.307	ns	-0.272	ns
Four seasons (n=48)	Body fat	-0.342	0.017	-0.055	ns	0.109	ns	0.114	ns	0.192	ns	0.230	ns	0.110	ns
	Glucose	-0.345	0.016	0.070	ns	0.240	ns	0.268	ns	0.011	ns	0.046	ns	-0.109	ns
	Leptin	-0.256	ns	0.100	ns	0.128	ns	0.309	ns	0.315	ns	0.339	0.043	0.275	ns
	CORT	-0.529	< 0.001	0.075	ns	0.337	0.044	0.371	0.026	0.227	ns	0.270	ns	0.167	ns

Note, Body fat: Total body fat mass; Glucose: Blood glucose; CORT: Corticosterone; ns: not significant

Biology Open • Supplementary information
